# Optic Nerve Sheath Diameter Remains Constant during Robot Assisted Laparoscopic Radical Prostatectomy

**DOI:** 10.1371/journal.pone.0111916

**Published:** 2014-11-04

**Authors:** Philip Verdonck, Alain F. Kalmar, Koen Suy, Thomas Geeraerts, Marcel Vercauteren, Alex Mottrie, Andre M. De Wolf, Jan F. A. Hendrickx

**Affiliations:** 1 Department of Anaesthesiology, University Hospital Antwerp, Edegem, Belgium; 2 Department of Anaesthesiology, University Medical Center Groningen, University of Groningen, Groningen, The Netherlands; 3 Department of Anaesthesiology and Intensive Care Medicine, OLV Clinic, Aalst, Belgium; 4 Department of Anaesthesiology and Critical care, University hospital of Toulouse, University Paul Sabatier, Toulouse, France; 5 Department of Urology, OLV Clinic, Aalst, Belgium; 6 Department of Anaesthesiology, Feinberg School of Medicine, Northwestern University, Chicago, Illinois, United States of America; 7 O.L.V. Vattikuti Robotic Surgery Institute, Melle, Belgium; University of Pennsylvania, United States of America

## Abstract

**Background:**

During robot assisted laparoscopic radical prostatectomy (RALRP), a CO_2_ pneumoperitoneum (CO_2_PP) is applied and the patient is placed in a head-down position. Intracranial pressure (ICP) is expected to acutely increase under these conditions. A non-invasive method, the optic nerve sheath diameter (ONSD) measurement, may warn us that the mechanism of protective cerebrospinal fluid (CSF) shifts becomes exhausted.

**Methods:**

After obtaining IRB approval and written informed consent, ONSD was measured by ocular ultrasound in 20 ASA I–II patients at various stages of the RALRP procedure: baseline awake, after induction, after applying the CO_2_PP, during head-down position, after resuming the supine position, in the postoperative anaesthesia care unit, and on day one postoperatively. Cerebral perfusion pressure (CPP) was calculated as the mean arterial (MAP) minus central venous pressure (CVP).

**Results:**

The ONSD did not change during head-down position, although the CVP increased from 4.2(2.5) mm Hg to 27.6(3.8) mm Hg. The CPP was decreased 70 min after assuming the head-down position until 15 min after resuming the supine position, but remained above 60 mm Hg at all times.

**Conclusion:**

Even though ICP has been documented to increase during CO_2_PP and head-down positioning, we did not find any changes in ONSD during head-down position. These results indicate that intracranial blood volume does not increase up to a point that CSF migration as a compensation mechanism becomes exhausted, suggesting any increases in ICP are likely to be small.

## Introduction

During robot assisted laparoscopic radical prostatectomy (RALRP), adequate surgical exposure requires the application of a CO_2_ pneumoperitoneum (CO_2_PP) and steep head-down position (up to 45°). The effects on the cardiopulmonary system are mild and well tolerated [1], [2], but those on intracerebral physiology remain poorly documented. According to the waterfall model, cerebral perfusion pressure (CPP) equals mean arterial pressure (MAP) minus central venous pressure (CVP) or intracranial pressure (ICP), whichever is higher [3]. Using only CVP and MAP measurements, CPP was found to remain within acceptable limits. In addition, although the sensitivity of near-infrared spectroscopy (NIRS) to detect cerebral ischemia is debatable, increases or slight decreases in cerebral oximetry readings during head-down position are reassuring [1], [4], [5].

The effect of ICP on CPP cannot easily be determined intraoperatively during RALRP, but in animal studies ICP has been shown to increase by up to 10 mm Hg above baseline with CO_2_PP and head-down position [6]–[11]. Transcranial Doppler measurement during RALRP revealed an increase in calculated zero flow pressure of equal magnitude as the measured increase in cerebral venous pressure, indicating that ICP does not exceed CVP [12]. More importantly, this increase in zero flow pressure did not further expand over the course of the operation, indicating no increase in cerebrovascular resistance and consequently no haemodynamically relevant increase in cerebral extracellular water content [1], [12].

When intracranial pressure varies within physiological limits, the consequences of the increase in ICP are restricted through compensatory mechanisms such as intracerebral blood and cerebrospinal fluid (CSF) shifts. It is only after exhausting these mechanisms that ICP would start to increase exponentially. Nevertheless, while permanent neurologic sequellae after robotic prostatectomy are rare in the absence of pre-existing intracranial pathology and effective CPP seems to be maintained within autoregulatory limits, severe complications have been reported [13], [14], which shows that it is possible at times these compensatory mechanisms are exhausted or near exhausted.

A new non-invasive method, the optic nerve sheath diameter (ONSD) measurement, may provide information about whether the mechanism of protective CSF fluid shifts to attenuate ICP increases becomes or is threatened to become exhausted in the individual patient undergoing RALRP.

Ocular sonography is safely used for ophthalmic evaluation since more than twenty years [15], and has a fast learning curve: novice sonologists need only 25 scans to obtain adequate results [16], with limited variability in measurement of ONSD, as median intra-observer and inter-observer variations were shown to be respectively less than 0.2 and 0.3 mm [17], [18], [19].

The CSF in the intracranial subarachnoidal space is connected to the CSF in the dural sheath around the optic nerve (ONS). Because CSF is incompressible, the ICP is directly transmitted to the fluid in the optic nerve sheath. Due to the elastic subarachnoidal trabecular anatomy, the optic nerve sheet is most distensible 3 mm behind the globe, thus making it the best point for ONSD interpretation.

The arachnoid surrounding the optic nerves approx. 3 mm proximal to the fovea has a highly ramified meshwork of delicate trabeculae [20]. The distensibilty of these trabeculae allows this chamber to inflate in case of raised ICP with an equilibration time of only a few minutes. In the ICU and emergency medicine literature, the ONSD was shown to correlate well with acute ICP changes [21]–[26]. The ONSD was reported to increase at CSF pressures between 15 and 30 mm Hg [21]. Inversely, a constant ONSD indicates that CSF pressure does not appreciably exceed these values. Reported cut-off values indicative of intracranial hypertension, defined as intracranial pressure above 20 mm Hg, vary from 5.0 mm to 5.9 mm, with a specificity of 86% and sensitivity of 79% [23]. Several recent clinical studies [17], [19], [22], [25] have compared sonographic ONSD with invasive *gold standard* methods for measuring ICP. Simultaneous measurements of ONSD and invasive ICP show a good relationship between both variables (r = 0.71 and r = 0.68) [19], [22], with similar cut-off values in acute neurocritical care patients. The best cutoff value was 5.7 or 5.8 mm for predicting elevated ICP (≥ or >20 mm Hg). The probability of having high ICP was very low (less than 5%) when ONSD was less than 5.8 mm. When comparing sonographic ONSD and ICP measured with a ventricular drain, the best cut-off value for detecting ICP>15 mm Hg (or 20 cm H_2_O) was 5 mm with a sensitivity and specificity of respectively 88% and 93% [22]. Moreover, changes in ONSD are strongly related to ICP variations (r = 0.73) [19], indicating that most probably, trends are even more indicative of relative changes in ICP than absolute values.

In patients undergoing RALRP, a constant ONSD would indicate that CSF migration as a compensation mechanism does not become exhausted, providing indirect evidence that ICP does not increase sufficiently to compromise cerebral perfusion. Because the ONSD was reported to increase at CSF pressures between 15 and 30 mm Hg [21], a constant ONSD also indicates that CSF pressure does not appreciably exceed these values. Because most patients awake uneventfully, and because our former observations showed that CPP values and cerebral oxygenation [1] remain within physiological ranges, we hypothesize that the ONSD does not change significantly during RALPR. In this study, the absence of significant changes in ONSD suggests that still an assuring safety margin in capacity for intracranial volume shift exists.

## Methods

After obtaining IRB approval and written informed consent, 20 ASA I–II patients undergoing RALRP were enrolled. Sample size calculation for our study was designed to detect an increase of 0.4 mm in ONSD between subsequent patient positioning. Measurements in 26 people with no disease and in 28 people with elevated intracranial pressure demonstrated a mean(SD) ONSD of 4.6(0.3) mm in normal adults versus 6.4(0.7) mm in the presence of increased ICP [27]. For an estimated SD of 0.5 mm between subsequent steady-state ONSD measurements, a power of 95% and an α-error of 5%, at least 19 patients should be included [28]. Therefore, we included 20 patients in total.

Patients were premedicated with 0.5 mg oral alprazolam one hour prior to surgery. After preoxygenation, anaesthesia was induced with sufentanil (0.1 µg/kg) and propofol (2–3 mg/kg). Intubation of the trachea was facilitated with rocuronium (0.6 mg/kg) and anaesthesia was maintained with sevoflurane at an end-expired concentration of 0.7–1 MAC in O_2_/air (F_I_O_2_ = 0.40) using a Zeus anaesthesia machine (Dräger, Lübeck, Germany). Ventilation using tidal volumes of 6–8 ml/kg with a maximal airway pressure of 25 cm H_2_O was adjusted to maintain the end-expired CO_2_ partial pressure (P_E_CO_2_) between 30 and 40 mmHg; positive end-expiratory pressure was not applied. The head was positioned in neutral position on a pillow designed to give additional support to the head and both shoulders to prevent the patient from sliding when placed in the head-down position. Head-down position was achieved by tilting the table to an angle range of 40° to 45°, adjusted to surgical exposure and laparoscopic accessibility.

Arterial blood pressure and CVP were measured in the left radial artery and right internal jugular vein, respectively. Both pressure transducers were positioned at the level of the external ear canal. Cerebral perfusion pressure (CPP), calculated as MAP minus CVP, was maintained above 60 mm Hg either by adjusting the sevoflurane concentration or by administering phenylephrine (100 µg boluses IV). Crystalloids (Hartmann, Braun) were administered as maintenance fluid, calculated for weight and additional third space losses. The insufflation pressure during the procedure was limited to 15 mm Hg. After anastomosis of the urethra, 20 mg furosemide was administered (per surgical protocol). Postoperatively, patients remained sedated with propofol and ventilated in the PACU by local protocol, to hasten turn-over, to diminish risk of airway difficulties (as sometimes seen after prolonged head-down position with pneumoperitoneum) and to have an extra measurement in supine position with a patient who is still sedated. One hour after arrival in the PACU, propofol administration was discontinued, the patients were allowed to awaken, and the trachea was extubated.

Ocular ultrasound measurements (GE Healthware Vivid.q) were made with a 13 MHz linear probe as previously described [21]. Eyelids were taped closed, and a thick layer of water-containing ultrasound gel was applied. The ONSD was measured at the entry zone of the optic nerve in the globe, 3 mm behind the papilla, perpendicular on the axis of the optic nerve. Transducer depth was set at 4 cm. ONSD measurements were made at 14 well defined moments during the procedure: before induction of anaesthesia (T0), 10 min after intubation, 10 min after insufflation, after 10–40–70–100–130–160–190 min of full Trendelenburg, 15–60 min after resuming supine position, awake in the PACU and one day postoperatively. All data were obtained by one of two observers (PV, KS). During each phase, the ONSD was measured once in the sagittal and once in the horizontal plane of each eye. The average of these 4 values was calculated. At those same moments, MAP, CVP and P_E_CO_2_ were recorded. To increase reproducibility, each image was reviewed by the second investigator, blinded for patient name and time point measured. Values are presented as mean (SD). Changes in MAP, CVP, CPP and P_E_CO_2_ values and differences of ONSD between measurements were analyzed with ANOVA for repeated measurements, followed by the Holm-Sidak test (SigmaPlot (Systat Software Inc, Vista Centre, Hounslow, London, UK)). Statistical significance level was set at 5%.

## Results

Reliable measurements could be performed in all patients. Data of the 20 patients were normally distributed. The age of the patients was 63(50–82) yr with a weight of 83(13) kg, a height of 177(6) cm and a body mass index of 26.6(3.7) kg m^−2^. Total fluid administration during the procedure was 1558(337) ml, total blood loss was 457(265) ml and total time in Trendelenburg position was 213(58) minutes. There were 9 patients with arterial hypertension and 1 with diabetes.

**Figure 1:**
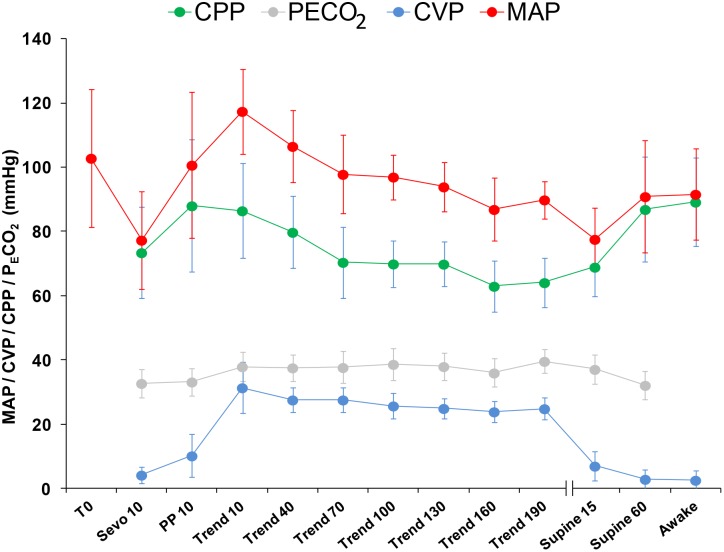
Evolution of physiological variables over the course of the procedure. Evolution of the mean (SD) values of the Cerebral Perfusion Pressure (CPP), End-tidal CO_2_ Pressure (P_E_CO_2_), Central Venous Pressure (CVP) and Mean Arterial Pressure (MAP) over the course of the procedure at different time points at 14 well defined moments during the procedure: before induction of anaesthesia (T0), 10 minutes after intubation, 10 minutes after insufflation, after 10–40–70–100–130–160–190 minutes of full Trendelenburg, 15–60 minutes after resuming supine position and awake at the PACU.

Figure 1 and table 1 show the evolution of MAP, CVP, CPP and P_E_CO_2_ over the course of the procedure. CPP increased from 73(14) mm Hg after intubation to 86(15) mm Hg in full Trendelenburg and decreased again to a lowest value of 63(8) mm Hg at 160 min of Trendelenburg. It normalised to 87(16) mm Hg at 60 min after resuming the supine position. CPP remained above 60 mm Hg at all times. The CVP increases from 4.2(2.5) mm Hg after intubation to a maximum of 27.6(3.8) mm Hg after 70 min of Trendelenburg. P_E_CO_2_ increased from 32.8(4.4) mm Hg after intubation (T_Sevo10_) to a maximum of 39.6(3.7) mm Hg after 190 min of Trendelenburg and CO_2_PP, and returned to baseline values 60 min after resuming the supine position. CPP, CVP and P_E_CO_2_ values changed significantly (p<0.05) between baseline and full Trendelenburg.

**Table 1:** Evolution of ONSD (mm), CPP (mm Hg) and P_E_CO_2_ (mm Hg) over the course of the procedure at 14 defined moments during the procedure: before induction of anaesthesia (T0), 10 minutes after intubation, 10 minutes after insufflation, after 10–40–70–100–130–160–190 minutes of full Trendelenburg, 15–60 minutes after resuming supine position, awake at the PACU (PA) and one day postoperatively (PO).

	ONSD		CPP		P_E_CO_2_	
	Mean	SD	Mean	SD	Mean	SD
T0	4.9	0.2	N/A	N/A	N/A	N/A
Sevo 10	4.9	0.3	73*	14	33*	4
PP 10	5.0	0.2	88*	21	33*	4
Trend 10	5.0*	0.3	87*	15	38	5
Trend 40	5.0*	0.3	80	11	38	4
Trend 70	5.0*	0.3	70**	11	38	5
Trend 100	5.0*	0.2	70**	7	39	5
Trend 130	5.0	0.2	70**	7	38	4
Trend 160	5.0	0.2	63**	8	36	4
Trend 190	5.1	0.2	64**	8	40	4
Supine 15	5.0	0.2	69**	9	37	5
Supine 60	4.9	0.2	87*	16	32*	4
Awake PA	4.8**	0.2	89*	14	0	0
Awake PO	4.8**	0.2	N/A	N/A	0	0

Values are shown in mean (SD).

ONSD: ** differs from * (except POD1 from T100).

CPP: ** differs from *.

P_E_CO_2_: * lower than other values.

**Figure 2:**
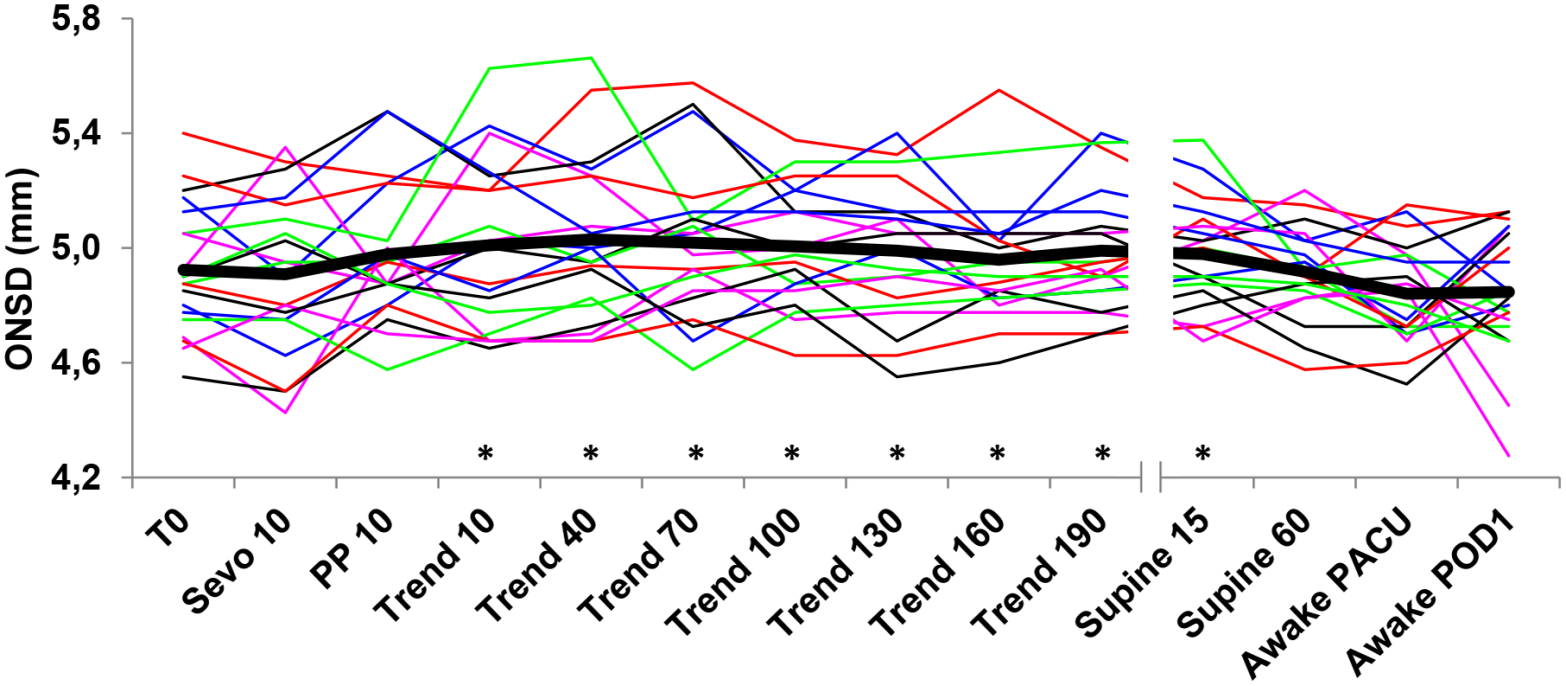
Evolution of ONSD over the course of the procedure. Evolution of the ONSD in individual patients (thin lines) and mean value (thick line) over the course of the procedure. *indicates significant differences with post-operative values.

The ONSD remained constant during the entire observation period (Figure 2 and table 1). All original data is freely available for reanalysis on request.

## Discussion

The ONSD does not change when patients undergoing RALRP are placed in a steep head-down position while a CO_2_PP is being applied. Previous studies in animals, as in humans suggest that applying a CO_2_PP and assuming the head-down position acutely increases ICP up to 10 mm Hg above baseline [9], [29]–[31]. However, since MAP and CVP increase to the same extent, and CVP is apparently higher than ICP, no net change in CPP should occur. CPP calculated as MAP – CVP has been documented to remain within the limits of cerebral blood flow autoregulation [3].

The hydrostatic pressure difference caused by the head down position causes both CVP and ICP to increase by the same amount relative to the pressure in the right atrium, but ICP could arguably still increase more than CVP. Institution of CO_2_PP and head-down position induces an increase in arterial blood volume and/or venous blood volume [3], [21]. The Monro-Kellie doctrine states that within the fixed and incompressible intracranial volume, its constituents (blood, CSF and brain tissue) impose a volume equilibrium where increases in volume of one compartment must be compensated by decreases in volume of the others [34]. As long as the increase in intracranial blood volume can be compensated by an equal decrease in CSF (by shifting the CSF out of the rigid container that the skull is), the ICP will not increase dramatically. Animal research has demonstrated that the normal mammal brain has a remarkable capacity to translocate CSF fluid to the vascular compartment [35]. The human brain can also translocate CSF very rapidly: to maintain ICP at 20 mm Hg, 2 mL min^−1^ of fluid has to be infused intrathecally [34]. Thus, an increase in intracranial blood volume can quickly be compensated by an efflux of CSF. Once the intracranial veins have reached their maximal dilatation, which was likely the case given the venous pressure of up to 27.6(3.8) mm Hg, no further increase in intracranial blood volume is to be expected. The Monro-Kellie doctrine therefore prompts us to examine whether in our patients sufficient CSF could be translocated to cushion this intracranial volume shift to prevent unacceptable increases in ICP and/or if these reserves are being exhausted (indicating cerebral tissue might be at risk of ischemia). Briefly, we expected that the combination of position and pneumoperitoneum would consistently raise ICP, and wanted to study if enough compensation mechanisms exist to keep this increase within acceptable limits. Our study reveals that *if* CSF translocation would be required to attenuate the increase in ICP in our patient population, this mechanism does not become exhausted up to a point where the ONSD increases. However, a significantly increased ICP due to positioning may be a rare event itself, given the relative rarity of complications consistent with an elevated ICP. Therefore, absence of ONSD increase in our results does not necessarily guarantee an equal safety zone in all patients.

In the patient undergoing RALRP in the presence of intracranial pathology with intracranial hypertension, or with a disturbed blood-brain barrier, head-down with a CO_2_PP *does* carry a risk: two patients undergoing radical cystectomy in head-down position during 7 and 10 hours respectively, were reported to have generalised cerebral edema, radiologically diagnosed after neurological deterioration in the PACU [36]. One of these patients had preexisting intracranial pathology. Consequently, an important limitation of our study is that in our patient population, strict exclusion of patients with a high a-priori risk for intracranial hypertension was respected, and therefore our reassuring conclusions should not be extrapolated to patients with pre-existing intracranial pathology.

While ONSD is not a perfect surrogate for ICP change, our current findings indicate that compensatory mechanisms including CSF translocation from the cranial vault to the spinal CSF compartment or the blood compartment are sufficient to attenuate ICP increases. Also pathology studies in animals have shown no evidence of tissue edema [32], [33]. Remarkably, another study with similar methodology and patient population to ours, did report a small but significant increase of ONSD of 12% during RALRP [37]. It has been suggested that some of the postoperative agitation seen in some of the patients might be related to the presence of some mild cerebral edema, but no evidence exist to either substantiate or refute this claim. MRI studies might be useful to help address this question.

Our findings support the existing claims that this steep patient positioning can be performed safely in a large patient population, and its risks should be balanced to the surgical advantages for optimal patient treatment. Importantly however, in mentioned previous studies, as in our current study, procedure duration was only moderate while duration of head -down position probably is a critical factor in generation of intracranial hypertension and possible cerebral edema, as already suggested by other authors [36]. More research is needed to determine the safety and effects on ICP of steep Trendelenburg positioning in longer lasting procedures, such as cystectomy. In addition, patients with intracranial pathologies were not included in our study, and our results should as such be restricted to patients without these conditions.

To summarise, RALRP requires a combination of a CO_2_PP and head-down positioning, which is known to increase intracranial pressure. The ONSD, previously validated as a means to help diagnose intracranial hypertension in the presence of head trauma, did not change in this setting. Our results in this study population with an apparent lack of intracranial pathology indicate that adequate compensation mechanisms may attenuate intracranial effects induced by the CO_2_PP and the head-down position, possibly by translocation of CSF towards the spinal canal and the vascular compartment. Additional research is needed to clarify the effects of prolonged head-down position in intracranial pressure in procedures of longer duration [38].

### Ethics statement

Our research protocol was approved by the “Ethisch Committee OLV Ziekenhuis, Aalst, Belgium” on January 5th 2012. After obtaining written informed consent, 20 ASA I–II patients undergoing RALRP were enrolled.

No animals were involved in this study.
