# Correction: Optic Nerve Sheath Diameter Remains Constant during Robot Assisted Laparoscopic Radical Prostatectomy

**DOI:** 10.1371/journal.pone.0118014

**Published:** 2015-02-03

**Authors:** The PLOS ONE Staff

There is a second affiliation for author Alain F. Kalmar that is not indicated. Alain F. Kalmar is also affiliated with: Department of Anaesthesiology and Intensive care medicine, Maria Middelares hospital, Ghent, Belgium.
